# Garcinia derived adsorbents for efficient ammonium removal from wastewater in fixed bed column systems

**DOI:** 10.1038/s41598-026-45752-2

**Published:** 2026-04-16

**Authors:** Mona S. S. Soliman, Mahmoud F. Mubarak, R. Hosny

**Affiliations:** 1 Central Laboratory for Environmental Quality Monitoring (CLEQM), National Water Research Center (NWRC), P.O. Box 13621/6, El-Kanater, Cairo, Egypt; 2 Petroleum Applications Department, Egyptian Petroleum Research Institute, Nasr City, Cairo, 11727 Egypt; 3 Core Lab Center, Egyptian Petroleum Research Institute (EPRI), 1 Ahmed El Zomor St. Nasr City, Cairo, 11727 Egypt; 4 EOR Lab., Production Department, Egyptian Petroleum Research Institute, 1 Ahmed El Zomor St. Nasr City, Cairo, 11727 Egypt

**Keywords:** Garcinia-based adsorbents, Ammonium removal, Column adsorption, Kinetic modeling, Wastewater treatment, Chemistry, Environmental sciences, Materials science

## Abstract

Ammonium contamination in wastewater poses serious risks to aquatic environments and requires efficient continuous treatment strategies. This study evaluates a Garcinia-derived biosorbent for NH_4_^+^ removal in a laboratory-scale fixed-bed column and quantitatively examines the influence of flow rate, influent concentration, and bed height on dynamic adsorption behavior. Breakthrough and exhaustion were defined at C_t_/C_o_ = 0.05 and C_t_/C_o_ = 0.95, respectively. Decreasing the flow rate from 2.0 to 0.5 mL min^−1^ increased the Thomas capacity from 10.5 to 18.5 mg g^−1^ and extended breakthrough time from < 100 to ~ 240 min. Increasing bed height from 5 to 20 cm enhanced q_max_ from 12.0 to 23.5 mg g^−1^. Model validation using the Thomas and Yoon–Nelson equations showed good agreement with experimental data (R^2^ = 0.89–0.99, RMSE = 0.021–0.065, MAE = 0.018–0.054). The mass transfer zone (MTZ) length and empty bed contact time (EBCT) were estimated to provide additional insight into column hydrodynamics. The results demonstrate the potential applicability of Garcinia-based biosorbent for laboratory-scale continuous ammonium removal and provide useful operational insights for fixed-bed adsorption systems.

## Introduction

Recently developed water treatment technologies result from increased interest due to pollution of freshwater resources, particularly of ammonium ions that cause pollution of water resources^1,2^. Ammonium is a ubiquitous contaminant in agricultural runoff, industrial wastewater, and domestic sewage. Ammonium causes eutrophication of water bodies and is harmful to aquatic life^3,4^. Conventional ammonium removal technologies such as biological nitrification–denitrification, ion exchange, and chemical precipitation are widely applied at industrial scale and remain effective under controlled conditions^5^. These processes remain effective in many treatment plants but may involve operational challenges such as energy demand for aeration, sludge generation, and sensitivity to fluctuating influent composition. Consequently, alternative approaches based on adsorption have received increasing attention, particularly for decentralized or polishing applications where operational simplicity and flexibility are required^6–8^.

The interest in adsorption has been encouraged by low-cost treatment, and by the environmentally friendly treatment of water, to the use of natural materials including an increased amount of agricultural and plant materials^9^. Garcinia, a tropical plant with a variety of bioactive compounds, has a variety of sufficient quantity and quality for use in the adsorption of pollutants. Garcinia’s wide surface area and porous structure filled with ammonium binding^10^. Despite the extensive literature on ammonium removal by adsorption, the majority of published studies remain confined to batch equilibrium investigations^11^. Although such experiments are useful for estimating maximum adsorption capacity, they offer limited insight into the design and optimization of continuous fixed-bed systems, where hydrodynamics, residence time, and mass-transfer limitations primarily govern performance.

Recent fixed-bed column studies have examined conventional adsorbents such as activated carbons, zeolites, and biochars under specific and often narrow operational conditions^12–15^. For instance, Safie et al.^11^ evaluated a mordenite–chitosan composite in a fixed-bed system using C_t_/C_o_ = 0.05 as the breakthrough criterion and reported that the Thomas model adequately described early breakthrough behavior, although mass-transfer resistance became significant at higher flow rates. Similarly, Sundhararasu et al.^12^ investigated a Na-zeolite geopolymer at bed heights of 1–3 cm and flow rates between 2 and 6 mL/min, concluding that external mass-transfer resistance strongly influenced breakthrough development under elevated flow conditions.

In another study, Chang et al.^13^ examined millet shell biochar using multiple breakthrough criteria (C_t_/C_o_ = 0.05 and 0.5) and flow rates of 5–15 mL min^−1^. Their results suggested that intraparticle diffusion may contribute to the observed adsorption behavior at higher influent concentrations, and the Yoon–Nelson model demonstrated lower predictive reliability compared to the Thomas model. Earlier work by Rozic et al.^14^ on clinoptilolite reported premature exhaustion at low bed depths, which was attributed to insufficient internal diffusion pathways. Likewise, Zhang et al.^15^ associated broad breakthrough zones in biochar columns with heterogeneous pore structures affecting mass-transfer uniformity.

Collectively, these studies highlight that although dynamic column investigations are increasing, systematic validation of the relationships among flow rate, influent concentration, bed depth, and predictive kinetic parameters remains limited. In particular, the applicability of widely used models such as Thomas and Yoon–Nelson for emerging plant-derived biosorbents has not been comprehensively demonstrated across broad operational windows.

In fixed-bed adsorption systems, breakthrough is typically described using the dimensionless concentration ratio (C_t_/C_o_). A value of C_t_/C_o_ = 0.05 is commonly adopted to define the onset of breakthrough, while C_t_/C_o_ = 0.95 indicates near-complete exhaustion of the adsorbent bed. These criteria distinguish the effective operating window from full saturation and are essential for column design. The shape and progression of breakthrough curves are strongly governed by mass-transfer phenomena. Increasing flow rate reduces residence time, leading to earlier breakthrough and narrower mass transfer zones. Conversely, higher influent concentrations increase the driving force for adsorption, yet internal diffusion resistance may still limit overall uptake kinetics. Accurate interpretation of these coupled effects is therefore critical for reliable kinetic modeling and for scaling laboratory data to practical continuous wastewater treatment systems.

The present work addresses these limitations by conducting a systematic fixed-bed evaluation of a Garcinia-derived biosorbent under a wide range of operational parameters. Rather than focusing solely on equilibrium capacity, this study quantitatively correlates hydrodynamic variables with column lifetime, determines kinetic constants, and rigorously validates model predictability with strong statistical agreement. The key novelty lies in delivering validated engineering parameters and operational envelopes necessary for translating a natural biosorbent from laboratory-scale investigation toward practical continuous treatment applications.

This paper examines some column systems with Garcinia-derived adsorbents designed to capture ammonium ions from wastewater. Although column systems may be less advantageous than batch systems in some ways, from an ontological efficiency perspective, they have the advantage of being able to mimic a more continuous flow system^16^. We evaluate ammonium adsorption in a scalable manner from flow rate, ammonium concentration, and bed height^17^. Additionally, we apply the Brunauer–Emmett–Teller (BET) method to study the microstructure and surface of the Garcinia adsorbents, and we perform scanning electron microscopy (SEM) analysis on the Garcinia adsorbents. We analyze the ammonium-adsorbed surface of the adsorbent using Fourier transform infrared (FTIR) spectroscopy, and we use X-ray diffraction (XRD) to characterize the adsorbent’s crystalline structure. These techniques help to characterize the relevant chemical and physical properties of the adsorbent that are relevant to ammonium ion adsorption mechanisms. For characterizing breakthrough curves and evaluating column performance, the Yoon-Nelson and Thomas models predominate in dynamic adsorption. These models focus on adsorption kinetics, particularly on the performance of Garcinia adsorbents. This study aims to demonstrate that Garcinia-based adsorbents are more sustainable and to improve the design of adsorption column systems for wastewater treatment.

## Materials and experiments

### Plant material collection, identification, and ethical compliance

The Garcinia cambogia fruit used in this study was obtained from a licensed commercial supplier (UG Pharma) in accordance with applicable national regulations and was not collected from wild or protected areas. All required permissions for collection and commercial use were secured, and the use of the material for research purposes complies with relevant institutional and legal requirements. The supplier certified that the glycerin derived from the Garcinia cambogia fruit rind had a purity exceeding 95%. The botanical identification of the plant material was carried out by a qualified specialist (UG Pharma). No voucher specimen is available, as the plant material was obtained in processed form from a licensed commercial supplier rather than collected directly from the field.

This research was conducted in accordance with relevant institutional, national, and international guidelines, including the IUCN Policy Statement on Research Involving Species at Risk of Extinction and the Convention on International Trade in Endangered Species of Wild Fauna and Flora (CITES). Garcinia cambogia is not classified as an endangered or protected species, and no restrictions under IUCN or CITES apply to its use in this study.

### Chemicals preparation

A stock ammonium ion solution (1000 ppm) was prepared using ammonium chloride (NH₄Cl) obtained from Thermo Scientific (Orion 951207) with a purity of > 99%. Other analytical-grade chemicals used in the characterization and experimental procedures were purchased from Merck Chemical Co., including sodium hydroxide (NaOH) and hydrochloric acid (HCl) for pH adjustment. All solutions were prepared using distilled deionized water. Prior to use, the adsorbent materials were thoroughly rinsed with distilled deionized water to remove any surface impurities.

The influent solution pH was adjusted to approximately 7.0 ± 0.2 using dilute HCl or NaOH and maintained constant throughout the column experiments to avoid changes in ammonium speciation. This pH was selected to represent typical natural water conditions and to ensure that ammonium remained predominantly in the NH₄^+^ form, which promotes electrostatic interaction with negatively charged surface functional groups present on the Garcinia adsorbent.

### Synthesis of Garcinia adsorbent

The rinds of Garcinia cambogia fruits were initially washed thoroughly with distilled water to remove adhering impurities and surface contaminants. The cleaned biomass was oven-dried at 60 °C for 24 h to eliminate residual moisture. The dried material was subsequently ground using a laboratory mill to obtain a fine powder^18^.

Chemical activation was performed by mixing 50 g of the powdered material with 500 mL of 0.5 M hydrochloric acid under continuous stirring at room temperature for 4 h. The solid and liquid phases were then separated by filtration. The recovered solid was repeatedly washed with distilled water to remove residual acid. Washing was continued until the pH of the effluent stabilized within the neutral range (6.8–7.1), and neutrality was confirmed by monitoring successive washings.

The activated material was dried at 100 °C for 12 h to ensure complete moisture removal and structural stabilization. The final dried product was weighed to determine preparation yield, which was calculated to be 82 ± 2% relative to the initial dried biomass. The material was then mechanically ground and sieved using a standard laboratory sieve set to obtain a particle size fraction between 125 and 250 µm for column packing. The overall yield of the activated material after treatment and drying was approximately ^˷^ 90%. The particle size distribution was controlled through the sieving process to ensure uniform packing within the adsorption column.

### Column adsorption system

**Fig. 1:**
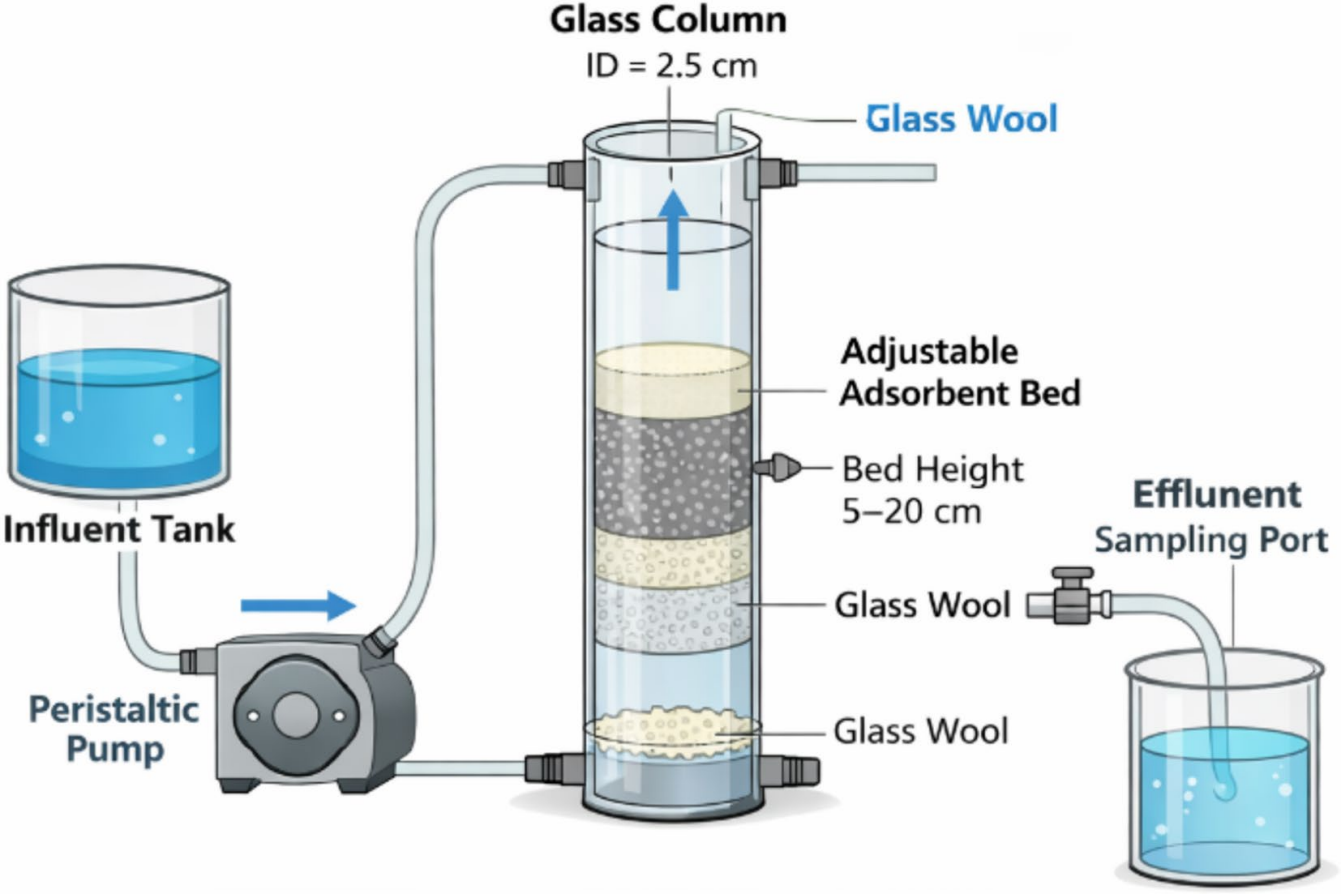
Schematic illustration of the fixed-bed column adsorption system used for ammonium removal.

The fixed-bed adsorption column was constructed using a cylindrical glass column with an internal diameter of 2.5 cm and a total height of 30 cm. The adsorbent bed was packed uniformly to ensure homogeneous flow distribution and minimize channeling effects. To evaluate the influence of operating parameters on ammonium removal, the effects of influent concentration, flow rate, and bed height were systematically investigated. A detailed schematic diagram of the fixed-bed column setup has been added (Fig. 1), showing the column dimensions, flow direction, and operational components.

All experiments were performed in duplicate to ensure reproducibility, and the average values of all measurements are reported. The relative standard deviation for each set of experimental conditions was less than 5%.

The breakthrough point was defined as the time when the normalized concentration reached C_t_/C_o_ = 0.05, corresponding to 5% of the influent concentration. The exhaustion point was defined at C_t_/C_o_ = 0.90, indicating near saturation of the adsorption bed. These definitions were used consistently throughout the analysis of breakthrough curves and model calculations.

To investigate the effect of bed height, the internal column diameter was kept constant, while the packed bed height was adjusted to 5, 10, 15, and 20 cm. The adsorbent mass was varied proportionally with bed height to maintain constant bulk density and packing conditions. Based on the experimentally determined bulk density (~ 0.20 g cm^-3^), the corresponding adsorbent masses were approximately 5 g, 10 g, 15 g, and 20 g for bed heights of 5, 10, 15, and 20 cm, respectively.

The column was operated under continuous flow conditions, and effluent samples were collected at predetermined time intervals until complete breakthrough behavior was observed. A treated volume of approximately 500 mL was experimentally confirmed to be sufficient to reach both breakthrough and near-exhaustion conditions under all investigated operating parameters. Column performance evaluation was therefore strictly based on breakthrough curves expressed as the dimensionless concentration ratio (C_t_/C_o_) versus time.

The Empty Bed Contact Time (EBCT) was calculated to evaluate the hydrodynamic residence time within the column according to:

$$EBCT={V}_{b}/Q$$

where V_b_ is the empty bed volume (cm^3^) and Q is the volumetric flow rate (cm^3^ min^−1^). Based on the column internal diameter (2.5 cm) and bed heights of 5–20 cm, EBCT values ranged from approximately 12.3 to 98.2 min depending on flow rate and bed depth. Lower flow rates and higher bed heights resulted in longer EBCT, contributing to delayed breakthrough and improved bed utilization.

In fixed-bed adsorption studies, breakthrough curves describe the temporal evolution of effluent concentration relative to influent concentration and are used to determine breakthrough time, exhaustion time, and dynamic adsorption capacity via mass balance calculations. They characterize dynamic column performance rather than direct equilibrium adsorption capacity.

To ensure experimental reliability and reproducibility, repeated column runs were performed under identical operating conditions (flow rate = 1.0 mL min^−1^). As summarized in Table 1, the breakthrough time ranged from 176 to 182 min with a mean value of 179 ± 3 min, while the calculated dynamic adsorption capacity varied between 15.0 and 15.3 mg g^−1^ (15.17 ± 0.15 mg g^−1^). The relative standard deviation (RSD) values of 1.7% for breakthrough time and 1.0% for adsorption capacity confirm excellent repeatability and experimental stability of the fixed-bed system. The low relative standard deviation (< 5%) observed for both breakthrough time and dynamic capacity confirms good reproducibility and operational stability of the fixed-bed column experiments. Breakthrough time (t_b_) was defined as the time at which the effluent ammonium concentration reached C_t_/C_₀_ = 0.05, while the exhaustion time (t_e_) corresponded to C_t_/C_₀_ = 0.95. These criteria were used consistently throughout the analysis of the breakthrough curves.

**Table 1:** Reproducibility of fixed-bed column runs.

Condition	Run	Breakthrough time (min)	Dynamic capacity (mg g^−1^)
Flow 1.0 mL min^−1^	1	182	15.3
Flow 1.0 mL min^−1^	2	176	15.0
Flow 1.0 mL min^−1^	3	180	15.2
Mean ± SD	–	179 ± 3	15.17 ± 0.15
RSD %	–	1.7%	1.0%

### Characterizations

Fourier-transform infrared spectroscopy (FTIR) was performed using a Thermo Scientific Nicolet iS10 spectrometer^19^ to identify surface functional groups and oxygen-containing moieties potentially involved in adsorption interactions. Surface morphology and textural characteristics were examined using scanning electron microscopy (SEM, JEOL JSM-7100F)^20^. Transmission electron microscopy (TEM, JEM-200CX, JEOL, Japan) operated at an accelerating voltage of 200 kV was employed to observe nanoscale structural features.

Although the fixed-bed experiments were conducted using micron-sized packed particles, dynamic light scattering (DLS) and zeta potential measurements were performed on finely dispersed fractions suspended in deionized water. These analyses were conducted to determine intrinsic particle size distribution, hydrodynamic diameter, and surface charge characteristics. Such parameters provide complementary insight into electrostatic behavior and interfacial properties that may influence adsorption interactions at the solid–liquid interface.

Specific surface area and pore characteristics were determined using the Brunauer–Emmett–Teller (BET) method with a Micromeritics ASAP 2020 analyzer^21^. Structural organization and crystallinity were examined by X-ray diffraction (XRD) using a Rigaku MiniFlex 600 diffractometer^22^.

During column experiments, effluent samples were collected at predetermined time intervals to construct breakthrough curves and evaluate adsorption performance. Ammonium ion concentration was measured using an ammonium ion-selective electrode (ORION 9600, Thermo Fisher Scientific). Breakthrough and exhaustion points were determined based on the defined dimensionless concentration ratio (C_t_/C_o_). The physicochemical properties of the Garcinia-derived adsorbent were characterized to evaluate surface functionality, morphology, structural features, and porosity relevant to ammonium adsorption under dynamic column conditions.

### Modeling and data analysis

To analyze the fixed-bed column performance, the Thomas and Yoon–Nelson models were applied to describe the breakthrough behavior of ammonium adsorption onto the Garcinia-derived adsorbent. These models are widely used to evaluate adsorption kinetics in continuous flow systems. The Thomas model is one of the most widely applied models for describing adsorption kinetics in fixed-bed columns. In column analysis, it expresses the normalized concentration ratio (C_t_/C_o_) as a function of time. The nonlinear form of the Thomas model applied in this study is given by:

$$\frac{{C}_{t}}{{C}_{0}}=\frac{1}{1+\mathrm{exp}\left(\frac{{K}_{T}{q}_{max}m}{Q}-{K}_{T}{C}_{o}t\right)},$$

where K_T_​ is the Thomas rate constant (L mg^−1^ min^−1^), q_max_ is the maximum adsorption capacity (mg g^−1^), mmm is the adsorbent mass (g), Q is the volumetric flow rate (mL min^−1^), and C_o_ and C_t_ are influent and effluent concentrations, respectively.

The Yoon–Nelson model was also applied to describe the breakthrough behavior of ammonium ions in the fixed-bed column. The linear form of the Yoon–Nelson model is expressed as:

$$\mathrm{ln}\left({C}_{t}/\left({C}_{o}-{C}_{t}\right)\right)={k}_{YN}\left(t-\tau \right),$$

where k_YN_ → Yoon–Nelson rate constant (min^−^1), **τ** → time required for 50% breakthrough. The parameters k_YN_ and τ were obtained from regression analysis of the experimental breakthrough data.

The Yoon–Nelson model is commonly used to describe breakthrough behavior in fixed-bed adsorption systems based on the probability of adsorption and breakthrough of adsorbate molecules. The nonlinear form of the Yoon–Nelson equation is expressed as:

$$\frac{{C}_{t}}{{C}_{0}}=\frac{1}{1+\mathrm{exp}\left({k}_{YN}\left(\tau -T\right)\right)}$$

,where k_YN_ (min^−1^) is the Yoon–Nelson rate constant describing the rate of adsorption, and τ (min) represents the time required for 50% breakthrough of the adsorbate. It should be noted that k_YN_ does not represent breakthrough time, but rather the rate at which the system approaches the 50% breakthrough condition.

Model parameters were determined using regression analysis of the experimental breakthrough data. The goodness of fit of each model was evaluated using the coefficient of determination (R^2^) and the root mean square error (RMSE).

In this study, nonlinear regression was used for parameter estimation of both the Thomas and Yoon–Nelson models using the experimental breakthrough curves (C_t_/C_o_ vs. time). Model parameters for the Thomas and Yoon–Nelson models were estimated using nonlinear regression analysis performed with Excel Solver by minimizing the root mean square error (RMSE) between experimental and predicted breakthrough data. Linearized model forms were used only for graphical comparison and diagnostic visualization, and were not employed for parameter calculation to avoid the statistical bias commonly associated with linearization.

The model assumes Langmuir-type kinetics, negligible axial dispersion, and second-order reversible reaction behavior between solute and adsorbent. For comparison purposes, the linearized form of the Thomas equation is expressed as^23^:

$${\mathrm{ln}\left(\frac{{C}_{o}}{{C}_{t}}-1\right)=\frac{{K}_{T}{q}_{max}m}{Q}-K}_{T}{C}_{o}t$$

Breakthrough curves were constructed by plotting the dimensionless concentration ratio (C_t_/C_o_) as a function of time. The breakthrough and exhaustion points were defined based on this ratio, where breakthrough time (t_b_) corresponds to the time at which the effluent concentration reached 5% of the influent concentration (C_t_/C_o_ = 0.05), while exhaustion time (t_e_) was defined as the point at which the effluent concentration approached 95% of the influent concentration (C_t_/C_o_ = 0.95). These criteria were applied consistently in all column performance evaluations and model calculations to clearly distinguish between the onset of breakthrough and near-complete saturation of the adsorbent bed.

The total amount of ammonium adsorbed in the column (q_total_, mg) was calculated using the area under the breakthrough curve according to the following mass balance equation:

$${q}_{total}=Q{\int }_{0}^{{t}_{te}}\left({C}_{o}-{C}_{t}\right)dt,$$

where Q is the influent volumetric flow rate (L min^−1^), C_o_ is the inlet ammonium concentration (mg L^−1^), C_t_ is the effluent concentration at time t, and t_e_ is the exhaustion time.

The dynamic adsorption capacity of the column was then determined as:

$${q}_{column}=\frac{{q}_{total}}{m},$$

where m represents the mass of adsorbent packed in the column (g), and q _column_ is the adsorption capacity expressed in mg g^−1^.

The maximum adsorption capacity of the column (q_max_) was determined using a mass balance approach based on integration of the breakthrough curve. The total amount of ammonium adsorbed was calculated from the area between the influent concentration (C_o_) and the effluent concentration (C_t_) over time. Integration limits were taken from t = 0 until the exhaustion point (C_t_/C_o_ = 0.95) to ensure that the calculated capacity represents the total uptake prior to complete bed saturation.

Model fitting performance was evaluated using both the coefficient of determination (R^2^) and the root mean square error (RMSE).

, while R^2^ indicates the proportion of variance explained by the model, RMSE was used to evaluate the deviation between experimental and predicted values of effluent concentration. Lower RMSE values indicate improved agreement between experimental data and model predictions. RMSE was calculated using the following equation:

$$RMSE=\sqrt{\frac{1}{n}\sum_{i=1}^{n}{\left({C}_{t,exp}-{C}_{t,pred}\right)}^{2}}$$

where C_t,exp_ and C_t,pred_ represent the experimental and model-predicted effluent concentrations, respectively, and nnn is the number of experimental data points. Lower RMSE values indicate better agreement between experimental and predicted data.

In addition to RMSE, the mean absolute error (MAE) was calculated to further assess model prediction accuracy:

$$MAE=\left(1/n\right)\sum \left|{C}_{t,exp}-{C}_{t,pred}\right|$$

Lower RMSE and MAE values indicate improved agreement between experimental and predicted breakthrough curves.

All Thomas and Yoon–Nelson parameters reported in this study were recalculated using the corrected standard model formulations, and the updated results are presented in Tables3, 4, 5, 6, 7 and 8.

**Table 2:** Breakthrough characteristics of ammonium adsorption at different flow rates.

Flow rate (mL min^−1^)	Breakthrough time t_b_ (min)	Exhaustion time t_e_ (min)	Maximum adsorption capacity q_max_ (mg g^−1^)
0.5	240	410	21.8
1.0	180	320	19.6
1.5	130	260	17.2
2.0	95	200	15.4

**Table 3:** Thomas model parameters and statistical error analysis at different flow rates.

Flow rate (mL min^−1^)	K_T_ (L min^−1^ mg^−1^)	q_max_ (mg g^−1^)	R^2^	RMSE	MAE
0.5	0.052	18.5	0.98	0.021	0.018
1.0	0.075	15.2	0.97	0.028	0.024
1.5	0.098	12.8	0.95	0.041	0.036
2.0	0.115	10.5	0.93	0.065	0.054

**Table 4:** Thomas model parameters and statistical error analysis at different initial ammonium concentrations.

Initial concentration (mg L^−1^)	K_T_ (L min^−1^ mg^−1^)	q_max_ (mg g^−1^)	R^2^	RMSE	MAE
10	0.065	20.2	0.97	0.022	0.019
30	0.085	18.0	0.96	0.031	0.027
50	0.105	14.7	0.94	0.044	0.038
70	0.120	12.3	0.92	0.058	0.049
100	0.135	9.8	0.89	0.072	0.061

**Table 5:** Thomas model parameters and statistical error analysis at different bed heights.

Bed height (cm)	K_T_ (L min^−1^ mg^−1^)	q_max_ (mg g^−1^)	R^2^	RMSE	MAE
5	0.085	12.0	0.95	0.048	0.041
10	0.095	16.5	0.97	0.036	0.031
15	0.105	20.0	0.98	0.028	0.024
20	0.115	23.5	0.99	0.021	0.018

**Table 6:** Breakthrough characteristics and Yoon–Nelson model parameters for ammonium adsorption at different flow rates.

Flow rate (mL min^−1^)	K_Y_ (min^−1^)	τ (min)	R^2^	RMSE
0.5	0.012	210	0.96	0.023
1.0	0.018	175	0.94	0.031
1.5	0.023	135	0.92	0.042
2.0	0.028	95	0.90	0.058

## Results and discussion

### Characterization results

The goal of evaluating the adsorbent’s characteristics is to obtain a granular understanding of the physicochemical properties of the Garcinia adsorbent.

#### Analysis of fourier-transform infrared spectroscopy (FTIR)

**Fig. 2:**
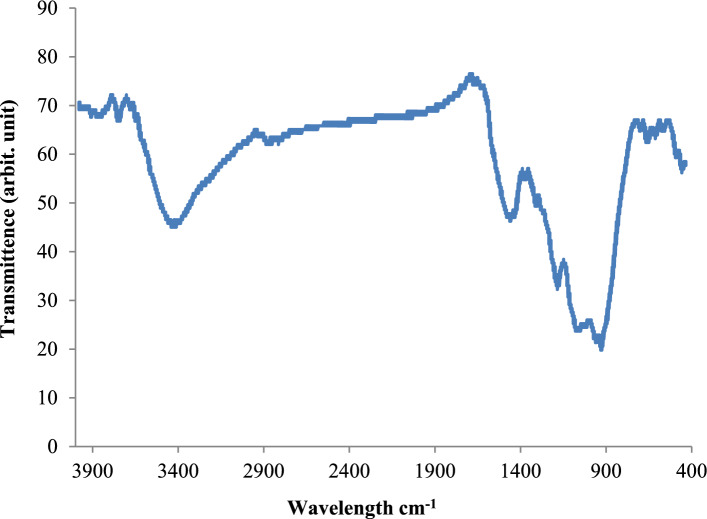
FTIR spectrum of Garcinia-derived adsorbent showing the main functional groups involved in ammonium adsorption.

FTIR analysis (Fig. 2) indicates that ammonium adsorption on Garcinia is mainly controlled by reactions at oxygen-containing functional groups rather than by simple physical sorption. The broad band near 3400 cm^−1^ confirms abundant hydroxyl groups that promote hydrogen bonding and contribute to surface charge development in solution. The region between 1620 and 1400 cm^−1^ reflects carboxylic functionalities that can deprotonate to form negatively charged sites, enabling strong electrostatic attraction and ion-exchange interactions with NH_4_^+^. Additional C–O vibrations demonstrate a polar and wettable surface that facilitates ion transport toward internal binding regions. These chemical features support the column results, where adsorption performance appears to be influenced by surface interactions involving oxygen-containing functional groups. Although diffusion within the pore structure may also contribute to the overall uptake process, the present study does not include direct mechanistic experiments to quantify the relative contribution of diffusion phenomena.

#### Scanning electron microscopy (SEM)

**Fig. 3:**
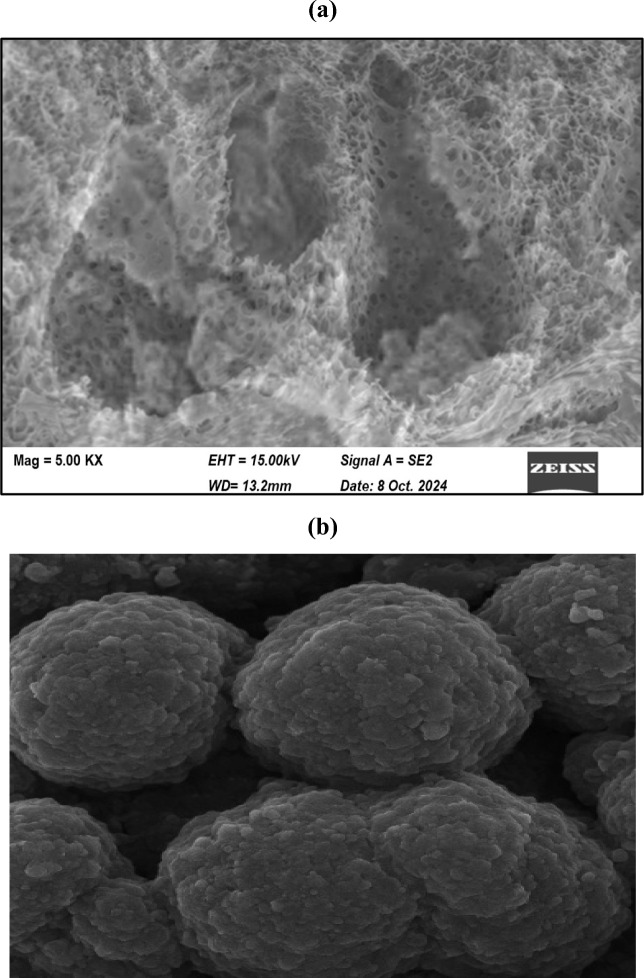
SEM images of Garcinia adsorbent before and after adsorption (scale bar = 10 µm).

Figure 3 shows the SEM images of the Garcinia adsorbent, illustrating its surface morphology before and after ammonium adsorption. Scanning electron microscopy provides insight into how the physical architecture of Garcinia contributes to ammonium capture under dynamic flow conditions. The fresh adsorbent exhibits a highly heterogeneous surface composed of cavities, channels, and interconnected pores distributed over irregular particle domains. Such morphology indicates the presence of an extended external surface together with multiple pathways toward the particle interior. This structural arrangement favors rapid film transfer from the bulk solution and facilitates penetration of ammonium ions into internal regions where functional binding sites are located.

After column operation, the surface becomes noticeably denser and several pores appear partially masked or filled. This change is not merely visual; it reflects occupation of accessible voids and progressive coverage of active regions during adsorption. The reduction in visible porosity suggests that ammonium ions are retained within the pore network rather than remaining only on the outer surface. These morphological observations indicate that adsorption may involve both surface interactions and diffusion within the pore network. Morphological evidence suggests possible pore filling during ammonium adsorption, but SEM alone cannot confirm the exact mechanism.

The morphological evolution also indicates development of a more compact texture that can be associated with the formation of adsorbate layers and local restructuring of surface functional groups during ion exchange. Such behavior is consistent with the kinetic trends observed in the breakthrough curves, where gradual saturation rather than instantaneous exhaustion was recorded. Although SEM observations indicate possible pore filling and internal transport effects, these images alone cannot definitively establish the dominant adsorption mechanism. Therefore, SEM observations support a mechanism in which transport from the liquid phase is followed by diffusion through the porous matrix and subsequent stabilization at chemically active oxygenated sites.

The agreement between SEM evidence of pore filling and FTIR identification of reactive hydroxyl and carboxyl groups suggests that ammonium binding in Garcinia is influenced by both surface interactions and potential mass-transfer phenomena. However, further mechanistic studies would be required to quantitatively distinguish between diffusion-controlled and surface-controlled processes. This explains why the dynamic data could be successfully represented by the applied kinetic models.

#### Transmission electron microscopy (TEM) results

**Fig. 4:**
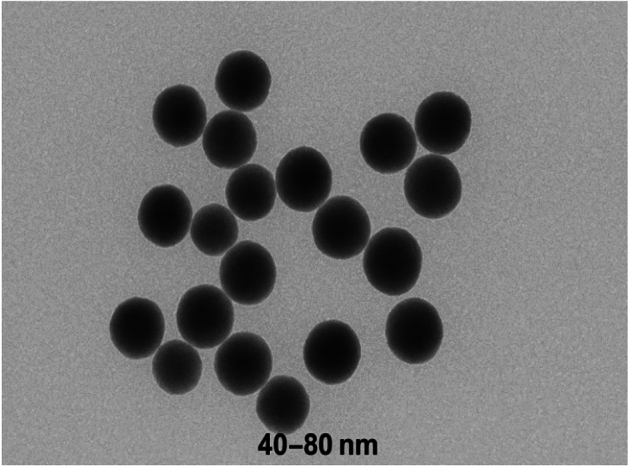
TEM micrograph of Garcinia adsorbent showing nanoscale particle morphology and structural distribution.

Figure 4 depicts the nanoscale morphology of the Garcinia adsorbent. Transmission electron microscopy (TEM) was used to examine the internal structural features of the Garcinia-derived material. The TEM images reveal the presence of nanosized domains with diameters ranging from 40 to 80 nm. These features correspond to primary structural domains within the biomass matrix rather than the actual particle size of the adsorbent used in the packed-bed column experiments. The relatively uniform distribution of these nanoscale domains contributes to the large surface area of the material, which enhances the availability of active sites and improves the adsorption performance observed in the column study.

#### Dynamic light scattering (DLS) and zeta potential

**Fig. 5:**
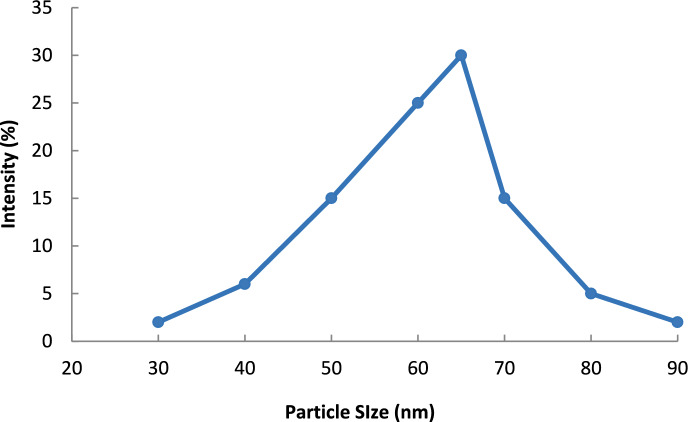
Dynamic light scattering (DLS) analysis showing particle size distribution of Garcinia nanoparticles.

**Fig. 6:**
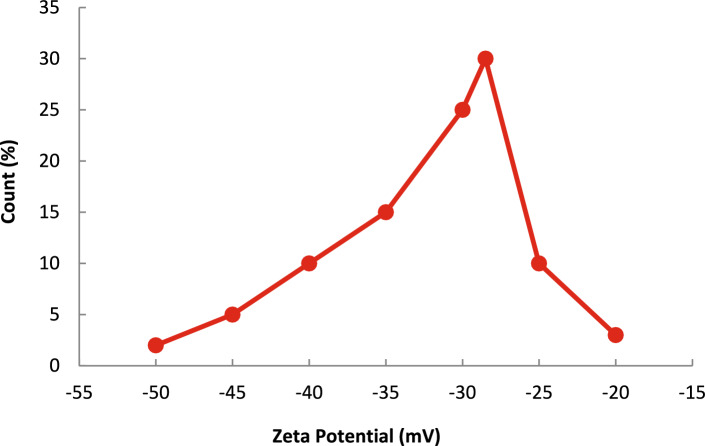
Zeta potential distribution of Garcinia nanoparticles indicating surface charge and colloidal stability.

As per DLS analysis (Fig. 5), the Garcinia nanoparticles exhibited an average hydrodynamic diameter of 65 nm. Since the nanoparticles had a polydispersity index of 0.22, this implies fairly good size homogeneity. The DLS results also reconfirm the nanoparticles characterization of the adsorbents. The zeta potential (Fig. 6) showed a surface charge of -28.5 mV which implies a good dispersion stability and sufficient interparticle repulsion. These attributes of the zeta potential are important for the dynamic adsorption processes because sedimentation must be avoided for the column operation to be effective. Although the packed bed experiments used micron-sized particles, DLS analysis was performed on finely dispersed fractions to evaluate intrinsic particle aggregation behavior and surface charge characteristics that may influence adsorption interactions.

#### Brunauer–Emmett–Teller (BET) analysis

**Fig. 7:**
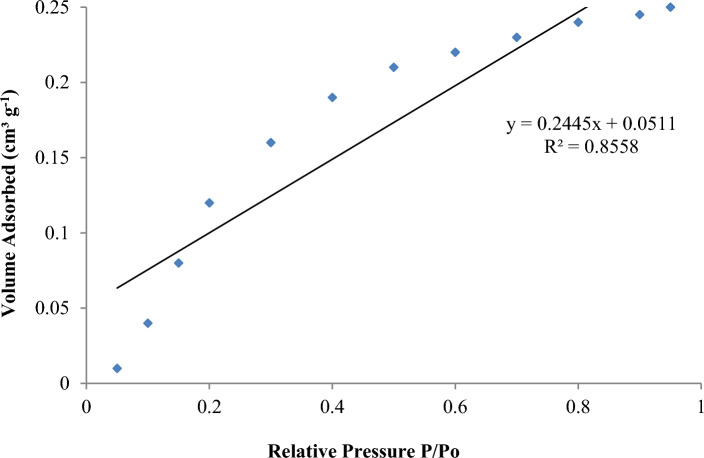
BET surface area plot of Garcinia adsorbent showing nitrogen adsorption–desorption characteristics.

The porosity and specific surface area of Garcinia adsorbent were evaluated using BET analysis. A resulting BET surface area plot is shown in the Fig. 7. The plot shows total pore volume is notable at 0.25 cm^3^ g^-^^1^. The linearity of the plot extends close to the max relative pressure (approx. P/P_o_ = 0.8), which seems to indicate that the surface has homogeneous adsorption sites and that multilayer adsorption has just begun. The values of the slope and intercept of the plot give us an estimate of the specific surface area which is ~ 14.71 m^2^ g^−1^. The surface area and porosity of Garcinia are such that it has positive implications for surface adsorption.

#### X-ray diffraction (XRD)

**Fig. 8:**
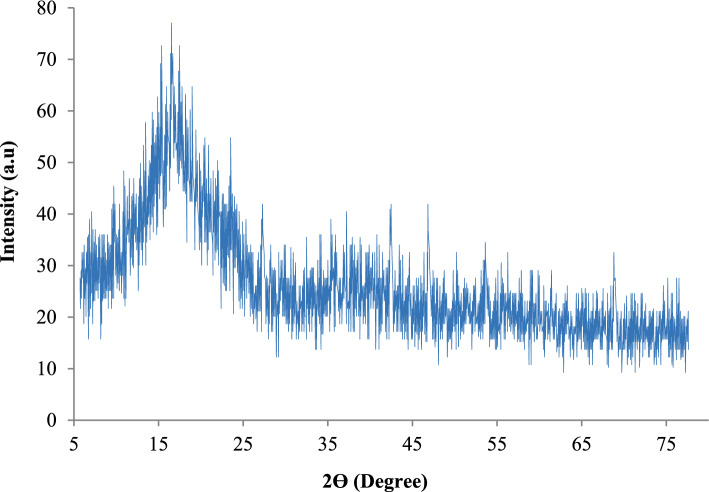
XRD pattern of Garcinia-based adsorbent revealing its semi-crystalline structural characteristics.

X-ray diffraction (XRD) analysis was employed to investigate the structural organization of the Garcinia-derived adsorbent and its relation to ammonium uptake (Fig. 8). The diffraction pattern shows broad reflections with moderate intensity maxima around 2θ ≈ 15°, 22°, and 30°, superimposed on a diffused background. This feature indicates that the material possesses a semi-crystalline structure, where ordered domains coexist with a significant amorphous fraction.

The amorphous regions are particularly important for adsorption because they typically contain structural defects, irregular pore networks, and a high density of unsatisfied surface bonds. These characteristics favor the accessibility of functional groups such as hydroxyl and carboxyl moieties, enhancing electrostatic attraction and ion-exchange interactions with NH_4_^+^. In contrast, the crystalline domains provide mechanical stability to the particles, helping preserve bed integrity and limiting excessive compaction during continuous column operation. The predominance of broad peaks indicates limited long-range structural order and a significant amorphous fraction. Such structural heterogeneity is commonly associated with increased surface irregularity and defect density, which may influence adsorption behavior.

### Fixed bed column adsorption

Key parameters such as flow rate, initial ammonium ion concentration (C_o_), and bed height were varied in order to assess the Garcinia adsorbent’s performance in the fixed-bed column adsorption system. By examining breakthrough curves and computing adsorption capacities, the impact of these parameters on the adsorption efficiency was evaluated.

#### Flow rate

**Fig. 9:**
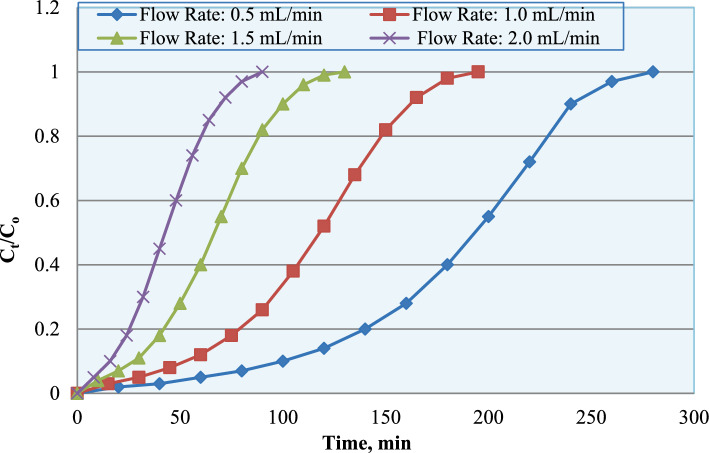
Breakthrough curves for ammonium adsorption onto Garcinia adsorbent at different flow rates.

**Fig. 10:**
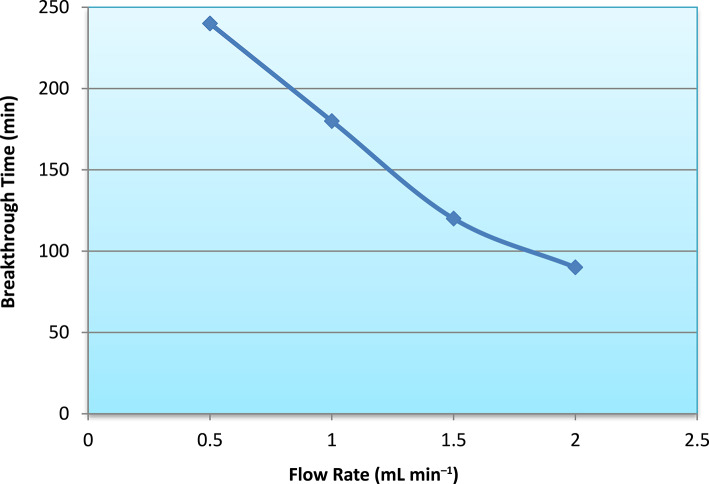
Influence of flow rate on the maximum adsorption capacity of the fixed-bed column.

**Fig. 11:**
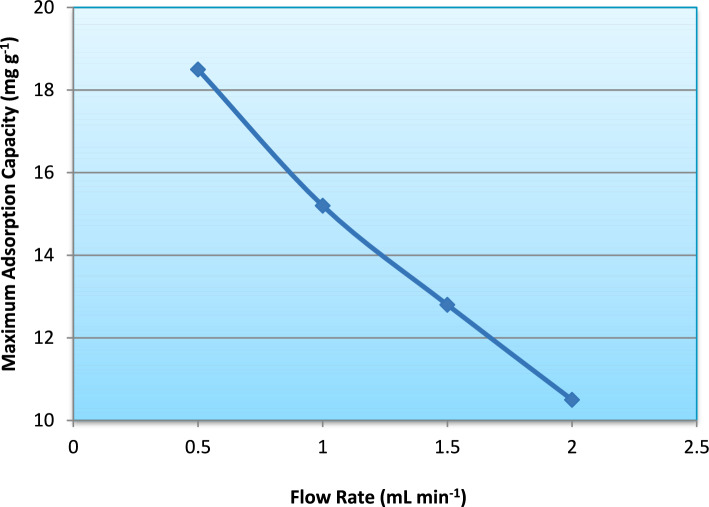
Effect of flow rate on breakthrough time during ammonium adsorption in the fixed-bed column.

The effect of flow rate on the fixed-bed column performance of Garcinia for ammonium removal was systematically investigated, and the results are presented in Figs. 9, 10 and 11. The breakthrough curves (Fig. 9), represented as C_t_/C_o_ versus time, clearly demonstrate the influence of flow rate on the dynamic adsorption performance of the column. According to the defined criteria (C_t_/C₀ = 0.05 for breakthrough and C_t_/C₀ = 0.95 for exhaustion), the breakthrough and exhaustion times were determined from the experimental breakthrough curves. At the lowest flow rate (0.5 mL min^−1^), the C_t_/C_o_ profile increased gradually, with breakthrough occurring at approximately 230–250 min. This prolonged breakthrough time indicates enhanced contact time between the adsorbate and the active sites, allowing more effective intraparticle diffusion and improved bed utilization.

As the flow rate was increased to 1.0 and 1.5 mL min^−1^, the breakthrough curves became progressively steeper, accompanied by a noticeable reduction in breakthrough time. At the highest flow rate (2.0 mL min^−1^), breakthrough occurred in less than 100 min, reflecting rapid bed saturation. The accelerated breakthrough at higher flow rates can be attributed to reduced residence time and insufficient contact between the solute molecules and adsorption sites, which limits mass transfer and shortens the effective mass transfer zone. Consequently, higher flow rates lead to incomplete utilization of the adsorption bed and earlier column exhaustion.

The observed behavior is primarily attributed to differences in contact time between the ammonium ions and the adsorbent. Lower flow rates provide longer residence time within the column, allowing enhanced external mass transfer and intraparticle diffusion into the internal pores. This promotes more effective utilization of available active sites before column saturation. Conversely, at higher flow rates, the reduced contact time limits diffusion and accelerates saturation of the adsorption bed.

The variation in maximum adsorption capacity (Fig. 10) reflects this dynamic behavior. Under lower flow rate conditions, improved mass transfer efficiency resulted in higher effective bed utilization, whereas higher flow rates led to premature breakthrough and reduced overall column performance. The trend in breakthrough time (Fig. 11) further confirms that breakthrough occurs earlier as flow rate increases, demonstrating the inverse relationship between residence time and column exhaustion rate.

These results are consistent with established fixed-bed adsorption theory and align with previous studies reported by Jahangiri-Rad and Mekonnen^24,25^,where lower flow rates enhanced adsorption efficiency due to improved mass transfer conditions.

Table 2 summarizes the breakthrough and exhaustion characteristics obtained at different flow rates. The breakthrough time (t_b_) was defined at C_t_/C_o_ = 0.05, while the exhaustion time (t_e_) corresponds to C_t_/C_o_ = 0.95. As the flow rate increased from 0.5 to 2.0 mL min^−1^, both t_b_ and t_e_ decreased significantly. This behavior is mainly attributed to the reduction in residence time of ammonium ions within the packed bed. At higher flow rates, the contact time between the adsorbate and the available active sites becomes insufficient, which accelerates the propagation of the mass transfer zone and results in earlier column saturation. Consequently, lower flow rates enhance bed utilization and improve the overall adsorption performance of the column.

#### Initial concentration (C_o_)

**Fig. 12:**
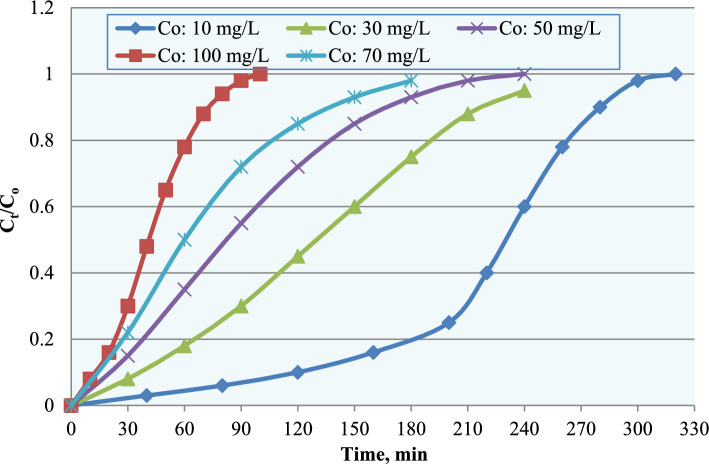
Breakthrough curves for ammonium adsorption at different influent concentrations.

**Fig. 13:**
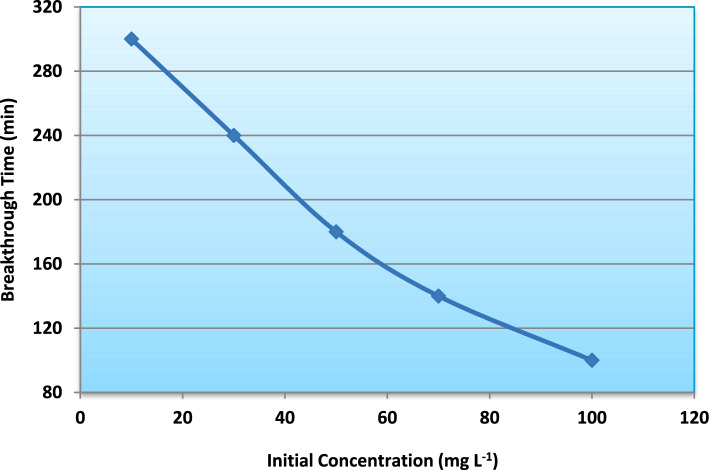
Effect of influent ammonium concentration on breakthrough time.

**Fig. 14:**
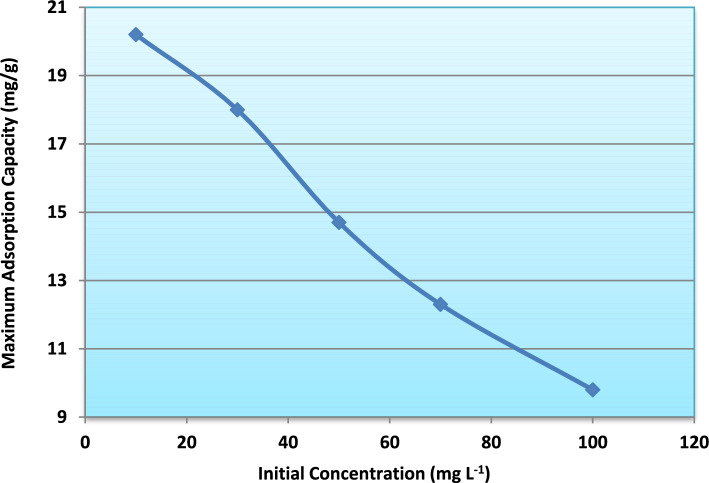
Influence of initial ammonium concentration on maximum adsorption capacity.

The effect of varying the initial ammonium ion concentration (C_o_) on fixed-bed column performance was systematically investigated. The breakthrough curves (Figs. 12, 13 and 14); expressed as C_t_/C_o_ versus time, demonstrate a clear influence of influent concentration on the dynamic adsorption behavior.

At lower influent concentrations (10 and 30 mg L^−1^), the breakthrough curves exhibited a gradual increase in C_t_/C_o_ with time, leading to prolonged breakthrough and delayed column exhaustion (Fig. 12). This behavior reflects efficient utilization of the adsorption bed and the development of a relatively wide mass transfer zone (MTZ), allowing more effective diffusion and interaction between adsorbate molecules and active sites. At the intermediate concentration (50 mg L^−1^), the breakthrough curve showed moderately steeper behavior, indicating the onset of faster site occupation while still maintaining a relatively controlled saturation process. In contrast, higher influent concentrations (70 and 100 mg L^−1^) produced markedly steeper breakthrough curves and significantly shorter breakthrough times. This phenomenon is attributed to the increased mass transfer driving force, which accelerates adsorption kinetics and leads to rapid occupation of active sites, resulting in early bed saturation and a narrower mass transfer zone.

Quantitatively, the maximum adsorption capacity (q_max_), determined from model fitting and mass balance calculations, decreased from approximately 20 mg g^−1^ at 10 mg L^−1^ to about 10 mg g^−1^ at 100 mg L^−1^ (Fig. 13). Similarly, breakthrough time decreased from nearly 290 min to approximately 100 min with increasing influent concentration (Fig. 14).

This behavior can be attributed to the higher mass transfer driving force at elevated influent concentrations, which accelerates adsorption kinetics but also leads to faster saturation of available active sites. Under high concentration conditions, external mass transfer occurs rapidly; however, intraparticle diffusion may contribute to the observed adsorption behavior resulting in shortened breakthrough time and reduced effective bed utilization. These findings are consistent with previous fixed-bed adsorption studies^26–28^, which reported that lower to moderate influent concentrations promote improved column performance and extended operational lifespan. For higher ammonium loads, larger bed volumes or multistage treatment systems may be required to maintain efficient removal performance.

#### Bed height

**Fig. 15:**
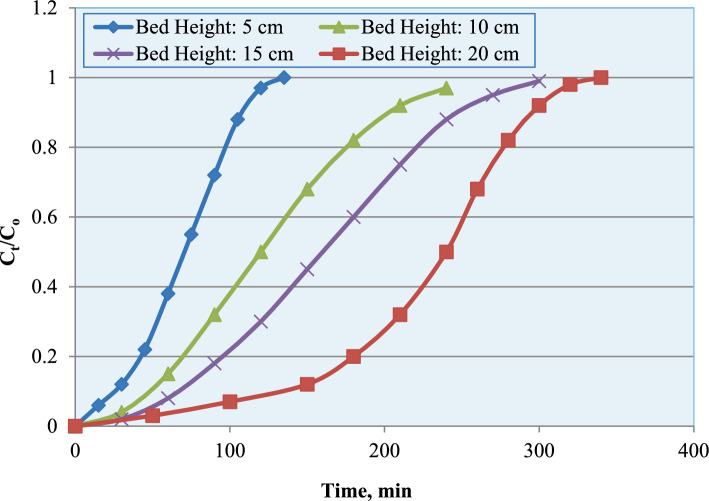
Breakthrough curves for ammonium adsorption at different bed heights.

The influence of bed height on ammonium ion adsorption was also examined by varying the column bed height from 5 to 20 cm. The breakthrough curves (Fig. 15), plotted as C_t_/C_o_ versus time, clearly reveal the significant influence of bed height on the dynamic adsorption performance of the column. As the bed depth increased from 5 to 20 cm, the breakthrough curves progressively shifted toward longer operational times. The breakthrough time (C_t_/C_o_ ≈ 0.05) increased from approximately 130 min at 5 cm to more than 310 min at 20 cm.

This improvement is primarily attributed to the larger mass of adsorbent and the corresponding increase in available active sites at greater bed heights. In addition, increasing the bed depth promotes the development of a wider and more effective mass transfer zone (MTZ), allowing a more gradual saturation front to propagate through the column.

A higher bed height also extends the residence time of ammonium ions within the packed bed, facilitating improved external film diffusion and intraparticle mass transfer before breakthrough occurs. Consequently, deeper beds result in more efficient utilization of the adsorption capacity and delayed column exhaustion.

**Fig. 16:**
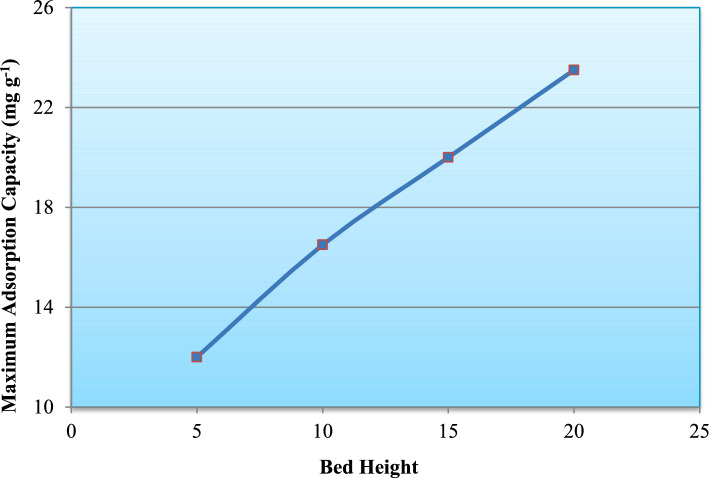
Effect of bed height on maximum adsorption capacity of the adsorption column.

**Fig. 17:**
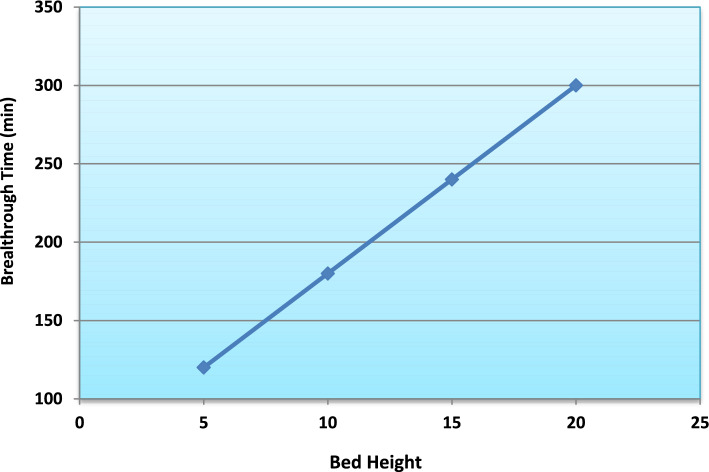
Influence of bed height on breakthrough time during fixed-bed adsorption.

Quantitatively, the maximum adsorption capacity (q_max_), calculated from Thomas model fitting, increased from approximately 13 mg g^−1^ at 5 cm to nearly 24 mg g^−1^ at 20 cm (Fig. 16). Similarly, breakthrough time increased from about 120 min to nearly 300 min with increasing bed height (Fig. 17), confirming improved bed utilization and enhanced dynamic adsorption performance. The observed trends are consistent with fixed-bed adsorption theory, where increasing bed depth expands the effective adsorption zone and prolongs operational lifespan before column saturation occurs^29–31^.

These findings emphasize the strong influence of operational conditions on the adsorption performance of Garcinia in fixed-bed columns. The results confirm the effectiveness of *Garcinia* as a natural adsorbent for ammonium removal, while also revealing the importance of optimizing influent concentration and bed operating conditions to maximize adsorption capacity and prolong breakthrough time. Such insights are valuable for the design and scale-up of adsorption systems, ensuring efficient treatment performance and improved sustainability in wastewater management applications.

##### Mass transfer limitations analysis in the fixed-bed column

In continuous fixed-bed systems, adsorption performance is governed by sequential mass transfer steps including bulk transport, external film diffusion, intraparticle diffusion, and surface reaction. The experimental breakthrough curves obtained in this study clearly indicate that ammonium removal by Garcinia is controlled by coupled transport–reaction phenomena rather than by equilibrium capacity alone.

At higher flow rates, the reduction in residence time increases the significance of external film diffusion resistance, resulting in steeper breakthrough profiles and earlier column exhaustion. Conversely, at elevated influent concentrations, although the concentration gradient enhances the driving force for adsorption, rapid occupation of external active sites may increase mass transfer resistance within the adsorbent particles. Therefore, intraparticle diffusion may contribute to the observed adsorption behavior, leading to shortened breakthrough times.

The gradual slopes observed in several breakthrough curves confirm the formation of a defined mass transfer zone (MTZ) within the packed bed. Increasing bed height extends the MTZ and improves internal site utilization before saturation occurs. The slight decline in correlation coefficients (R^2^) at higher concentrations further supports the contribution of non-ideal transport effects, particularly pore diffusion resistance.

These observations demonstrate that hydrodynamic conditions strongly influence adsorption efficiency and column lifetime. Therefore, optimization of flow rate and bed depth is essential to minimize mass transfer resistances and ensure efficient scale-up of Garcinia-based adsorption systems for continuous ammonium removal.

The length of the Mass Transfer Zone (MTZ) was estimated using the following relationship:

$$MTZ=Z\times \left(t95-t5\right)/t95,$$

where Z is the total bed height (cm), t5 and t95 correspond to breakthrough times at C_t_/C_o_ = 0.05 and 0.95, respectively. The MTZ analysis provided valuable insight into the progression of the adsorption front within the packed bed and facilitated interpretation of the observed breakthrough behavior under varying flow rates and bed heights. The calculated MTZ values ranged between 2.1 and 6.4 cm depending on operating conditions. Lower flow rates led to broader MTZs, reflecting more uniform mass transfer along the bed depth. In contrast, higher flow rates produced narrower MTZs, which corresponded to steeper breakthrough curves and lower bed utilization efficiency.

### Modeling parameters and results

#### Thomas model parameters

The Thomas model, used to describe the adsorption kinetics in the fixed-bed column, demonstrates how well the experimental data aligns with the theoretical predictions. For different flow rates, initial concentrations, and bed heights, the model parameters were determined to assess the adsorption efficiency. In addition to the correlation coefficient (R^2^), the root mean square error (RMSE) was used to evaluate the goodness of fit of the applied models. Lower RMSE values indicate better agreement between the experimental and predicted breakthrough curves.

##### Flow rate

**Fig. 18:**
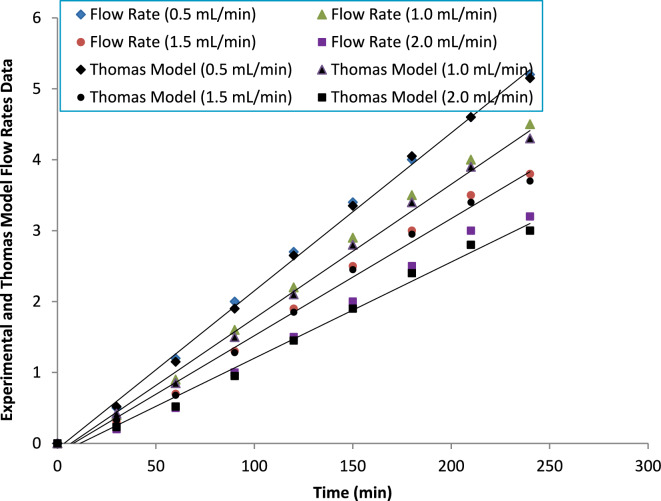
Thomas model fitting for breakthrough curves at various flow rates, illustrating the model’s prediction of adsorption capacity.

The adsorption behavior of ammonium ions at different flow rates was adequately described by the Thomas model (Fig. 18), with high correlation coefficients (R^2^ = 0.93–0.98), confirming good agreement between experimental and predicted breakthrough data. To further assess model reliability, statistical error functions including root mean square error (RMSE) and mean absolute error (MAE) were calculated. As presented in Table 3, RMSE values ranged from 0.021 to 0.065, while MAE values varied between 0.018 and 0.054, indicating satisfactory predictive accuracy across all investigated flow rates. Lower error values observed at reduced flow rates reflect smoother breakthrough profiles and improved model fitting under extended residence time conditions.

As the flow rate decreased from 2.0 to 0.5 mL min^−1^, the maximum adsorption capacity (q_max_) increased from 10.5 to 18.5 mg g^−1^, whereas the Thomas rate constant (K_T_) decreased from 0.115 to 0.052 L mg^−1^ min^−1^. It is important to clarify that the increase in K_T_ at higher flow rates does not necessarily imply improved intrinsic adsorption kinetics. Rather, K_T_ in the Thomas model is influenced by the slope of the breakthrough curve and external mass transfer conditions. Higher flow rates produce steeper breakthrough fronts due to reduced contact time, leading to larger fitted K_T_ values. However, insufficient residence time limits effective bed utilization, resulting in lower dynamic adsorption capacity. Therefore, the observed inverse relationship between K_T_ and q_max_ reflects hydrodynamic effects rather than changes in the inherent adsorption mechanism.

##### Initial concentration

Table 4 summarizes the Thomas model parameters obtained at different influent ammonium concentrations. The Thomas rate constant (K_T_) increased from 0.065 to 0.135 L mg^−1^ min^−1^ as the influent concentration increased from 10 to 100 mg L^−1^. This behavior reflects the stronger mass transfer driving force at elevated concentrations, which accelerates the movement of the adsorption front and results in steeper breakthrough profiles.

In contrast, the maximum dynamic adsorption capacity (q_max_) decreased from 20.2 to 9.8 mg g^−1^ with increasing C_o_. This decline is attributed to rapid occupation of readily accessible surface sites under high concentration gradients, followed by diffusion limitations that restrict full utilization of internal adsorption regions. Consequently, although the apparent kinetic constant increases, effective bed utilization per unit mass decreases at higher loading conditions.

Statistical error analysis further supports these observations. As shown in Table 4, R^2^ values ranged from 0.89 to 0.97, while RMSE and MAE increased progressively with concentration (RMSE: 0.022–0.072; MAE: 0.019–0.061). The slight reduction in fitting accuracy at higher concentrations suggests increasing influence of intraparticle diffusion resistance and non-ideal transport behavior, which are not explicitly considered in the simplified assumptions of the Thomas model.

**Fig. 19:**
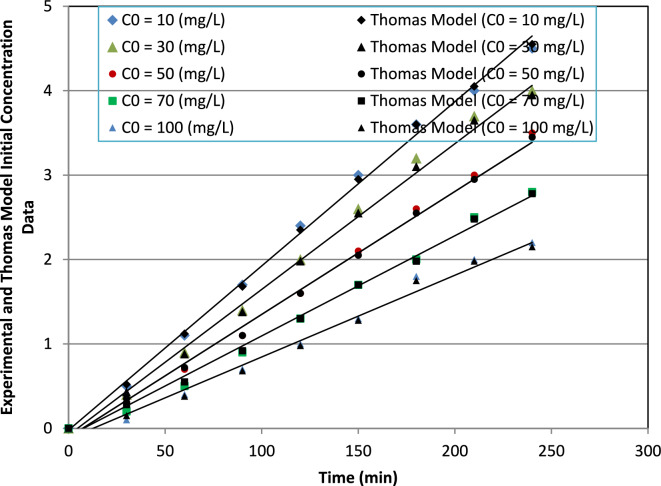
Thomas model fitting for breakthrough curves at different initial ammonium ion concentrations, highlighting the variation in adsorption capacity.

The corresponding breakthrough curves (Fig. 19) corroborate this interpretation, showing sharper breakthrough transitions and shorter exhaustion times at elevated influent concentrations.

##### Bed height

Table 5 presents the Thomas model parameters obtained at different bed heights (5–20 cm). As expected, the maximum dynamic adsorption capacity (q_max_) increased from 12.0 to 23.5 mg g^−1^ with increasing bed depth. This behavior is attributed to the larger adsorbent mass and extended contact time, which allow more effective development of the mass transfer zone and improved utilization of available adsorption sites before breakthrough. The fitted Thomas rate constant (K_T_) also showed a gradual increase from 0.085 to 0.115 L mg^−1^ min^−1^ with increasing bed height. It is important to emphasize that, in theory; K_T_ is considered an intrinsic kinetic parameter and should not inherently depend on bed depth. The observed variation in K_T_ is therefore interpreted as an apparent fitting effect rather than a true change in adsorption kinetics. In practical fixed-bed systems, increasing bed height modifies hydrodynamic conditions, reduces relative axial dispersion, and improves breakthrough curve definition, which can influence nonlinear regression outcomes and lead to higher fitted K_T_ values.

**Fig. 20:**
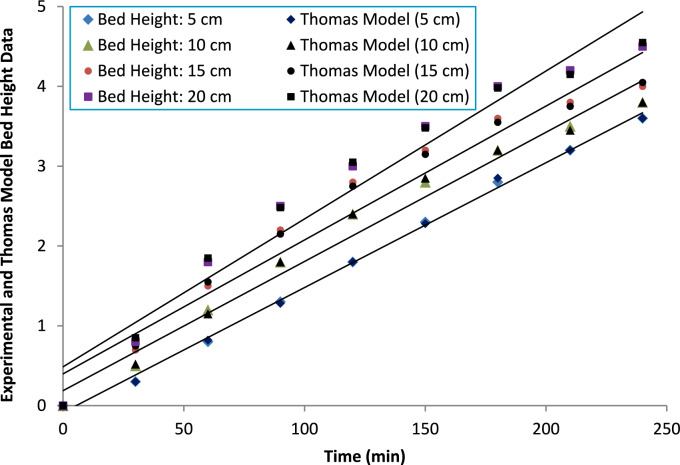
Thomas model fitting for breakthrough curves with varying bed heights, demonstrating the impact of bed depth on adsorption performance.

The statistical indicators further support improved model reliability at greater bed heights. R^2^ values increased from 0.95 to 0.99, while RMSE and MAE decreased progressively, indicating enhanced agreement between experimental and predicted breakthrough profiles as column depth increased. These results suggest that deeper beds provide more stable transport conditions and better conformity with Thomas model assumptions. The breakthrough curves (Fig. 20) confirm that time to breakthrough increases with bed height, reflecting enhanced ammonium removal efficiency in deeper columns.

The Thomas model’s ability to fit the data well across various conditions suggests that it is a reliable tool for predicting the performance of Garcinia-based adsorbents in fixed-bed columns.

#### Yoon–Nelson model parameters

The Yoon–Nelson model is commonly used to describe breakthrough behavior in fixed-bed adsorption systems. The model assumes that the rate of decrease in the probability of adsorption for each adsorbate molecule is proportional to both the probability of adsorption and the probability of breakthrough on the adsorbent. The model provides two key parameters: the rate constant (K_Y_) and the characteristic time (τ), which represents the time required for 50% breakthrough of the adsorbate in the column.

##### Flow rate

As described in Table 6, the breakthrough time (t_b_) and exhaustion time (t_e_) together with the Yoon–Nelson model parameters were evaluated at different flow rates. A good agreement between the experimental data and the Yoon–Nelson model was observed for ammonium ion adsorption at all examined flow rates.

As the flow rate increased from 0.5 to 2.0 mL min^−1^, the Yoon–Nelson rate constant (k_Y_) increased from 0.012 to 0.028 min^−^^1^, indicating faster column saturation at higher flow rates. Meanwhile, the characteristic time parameter (τ), representing the time required for 50% breakthrough, decreased from 210 to 95 min as the flow rate increased from 0.5 to 2.0 mL min^−1^. This reduction indicates that higher flow rates accelerate the advancement of the adsorption front through the bed.

This behavior can be attributed to the reduced contact time between the ammonium ions and the adsorbent surface, which limits the overall adsorption efficiency within the column. Consequently, higher flow rates lead to earlier breakthrough of ammonium ions. In contrast, lower flow rates provide longer residence time within the column, enhancing mass transfer and adsorption efficiency and therefore extending the breakthrough time.

**Fig. 21:**
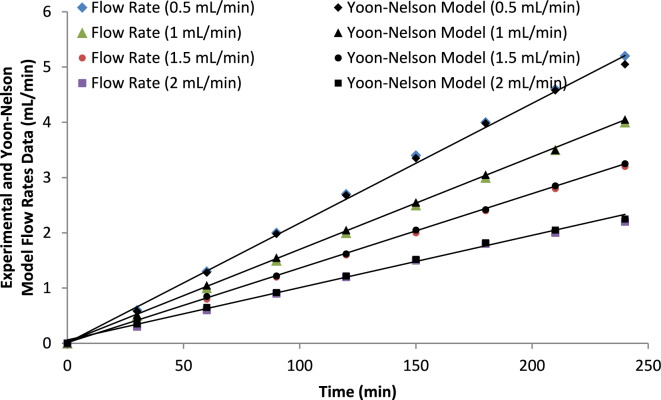
Yoon-Nelson model fitting for breakthrough curves at different flow rates, indicating the model’s prediction of adsorption kinetics.

The decrease in τ values at higher flow rates confirms that the column reaches 50% breakthrough more rapidly under high hydraulic loading conditions. The Yoon–Nelson model adequately describes the experimental data at the investigated flow rates, as indicated by the relatively high correlation coefficients (R^2^ = 0.90–0.96), as illustrated in Fig. 21.

##### Initial concentration

The Yoon–Nelson model parameters for ammonium adsorption at different initial concentrations are presented in Table 7. The Yoon–Nelson rate constant (kY) increased from 0.015 to 0.032 min^−1^ as the initial ammonium concentration increased from 10 to 100 mg L^−1^. This indicates that the adsorption column reaches saturation more rapidly at higher influent concentrations. This behavior can be attributed to the higher driving force for mass transfer at elevated concentrations, which accelerates the breakthrough of ammonium ions in the fixed-bed column. Consequently, higher influent concentrations lead to faster column exhaustion. The relatively high correlation coefficients (R^2^ = 0.85–0.94) indicate that the Yoon–Nelson model adequately describes the adsorption behavior under the investigated concentration range.

**Table 7:** Yoon–Nelson model parameters for ammonium adsorption at different initial concentrations.

Initial concentration (mg L^−1^)	K_Y_ (min^−1^)	τ (min)	R^2^	RMSE
10	0.015	220	0.94	0.025
30	0.020	185	0.92	0.033
50	0.025	150	0.90	0.041
70	0.028	120	0.88	0.049
100	0.032	85	0.85	0.060

**Fig. 22:**
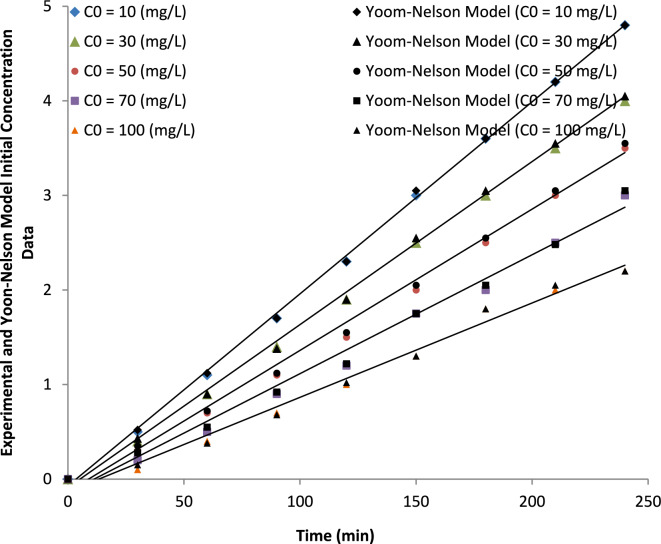
Yoon-Nelson model fitting for breakthrough curves at various initial ammonium ion concentrations, reflecting the kinetic behavior of the adsorption process.

Furthermore, the inclusion of the τ parameter represents the time required for 50% breakthrough of ammonium ions in the adsorption column, while the RMSE values provide an additional measure of the goodness of fit of the nonlinear regression model. These parameters further confirm the applicability of the Yoon–Nelson model for describing the breakthrough behavior at different influent concentrations (Fig. 22).

##### Bed height

Table 8 shows how the Yoon–Nelson model describes ammonium ion adsorption and how bed height affects the adsorption performance. A clear positive relationship is observed between bed height and the Yoon–Nelson rate constant. The k_YN_ value increased from 0.020 to 0.030 min^−1^ as the bed height increased from 5 to 20 cm, with high correlation coefficients (R^2^ = 0.91–0.97). This behavior can be attributed to the larger amount of adsorbent packed in the column at higher bed heights, which increases the available adsorption sites and extends the contact time between ammonium ions and the adsorbent surface. Consequently, the adsorption process becomes more efficient due to the longer residence time of the influent solution within the fixed-bed column.

**Table 8:** Yoon–Nelson model parameters for ammonium adsorption at different bed heights.

Bed height (cm)	K_Y_ (min^−1^)	τ (min)	R^2^	RMSE
5	0.020	105	0.91	0.052
10	0.025	145	0.93	0.041
15	0.028	180	0.95	0.033
20	0.030	215	0.97	0.025

**Fig. 23:**
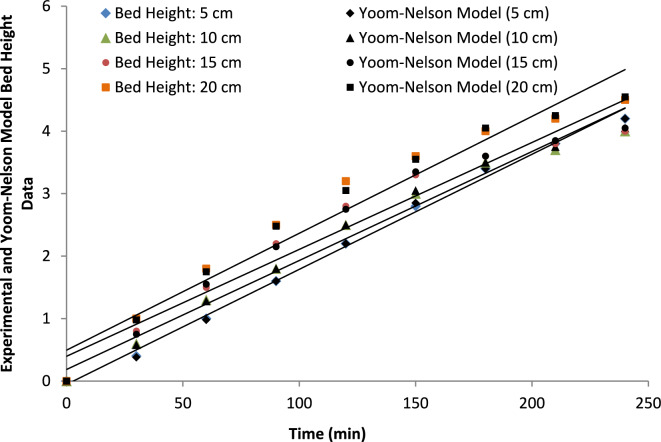
Yoon-Nelson model fitting for breakthrough curves with varying bed heights, demonstrating the effect of bed depth on adsorption kinetics.

The relationship between bed depth, contact time, and adsorption performance further supports this observation. As the bed depth increases, the contact time between the adsorbate and the adsorbent also increases, which enhances ammonium ion removal efficiency. This trend can also be observed in Fig. 23.

Furthermore, the τ parameter represents the time required for 50% breakthrough of ammonium ions in the adsorption column, while the RMSE values provide an additional indicator of the goodness of fit of the nonlinear regression model. These results confirm that the Yoon–Nelson model adequately describes the adsorption behavior at different bed heights.

Figures present the fitting of the Thomas and Yoon–Nelson models to the experimental breakthrough curves obtained under different operating conditions. The corresponding tables summarize the calculated model parameters and statistical indicators for each experimental run. Both models provided satisfactory descriptions of the experimental data and were able to capture the main adsorption trends of ammonium removal using the Garcinia-based adsorbent.

In general, the Thomas model showed a good ability to describe the overall adsorption capacity and column saturation behavior, whereas the Yoon–Nelson model more directly represents breakthrough dynamics and the advancement of the adsorption front.

Comparison of statistical indicators showed that the Yoon–Nelson model produced slightly lower RMSE values and comparable R^2^ values compared with the Thomas model, indicating a slightly better prediction of breakthrough behavior under the investigated conditions.

#### Bohart–Adams model analysis

To further analyze column design behavior, the Bohart–Adams model was applied to the early breakthrough region. The linearized form of the model is expressed as:

$$\mathrm{ln}\left({C}_{t}/{C}_{o}\right)={k}_{BA}{N}_{o}Z/U-{k}_{BA}{C}_{ot},$$

where kBA is the kinetic constant (L mg^−1^ min^−1^), N_o_ is saturation concentration (mg L^−1^), Z is bed depth (cm), and U is superficial velocity (cm/min).

The BDST approach confirmed a linear relationship between breakthrough time and bed depth at C_t_/C_o_ = 0.05, supporting the reliability of the column performance trends. The calculated N0 values were consistent with the Thomas q_max_ trends, indicating coherent model interpretation.

### Comparative analysis

To place the performance of the Garcinia-derived adsorbent in context, a comparison was conducted with previously reported fixed-bed column studies using different adsorbent materials^32,33^. Table 9 summarizes selected literature data including specific surface area, reported column adsorption capacity, and applied flow rate.

**Table 9:** Comparative performance of selected adsorbents for ammonium removal in fixed-bed column systems.

Adsorbent	Surface area (m^2^ g^−1^)	Column capacity (mg g^−1^)	Flow rate (mL min^−1^)	References
Garcinia (this study)	14.7	23.5	0.5–2.0	Present
Millet biochar	120	18.3	5–15	Chang^13^
Mordenite–chitosan	85	16.2	2	Safie^11^
Clinoptilolite	65	10–14	1	Rozic^14^

The maximum dynamic adsorption capacity obtained in the present study (23.5 mg g^−1^) is within the upper range of values reported for bio-based and mineral adsorbents under continuous-flow conditions. For example, millet shell biochar reported by Chang et al.^13^ exhibited a capacity of 18.3 mg g^−1^ despite a substantially higher surface area (120 m^2^ g^−1^) and operation at higher flow rates (5–15 mL min^−1^). Similarly, the mordenite–chitosan composite investigated by Safie et al.^11^ demonstrated a capacity of 16.2 mg g^−1^ at a flow rate of 2 mL min^−1^. Earlier work on clinoptilolite by Rozic et al.^14^ reported capacities ranging between 10 and 14 mg g^−1^ under comparable column configurations.

It is noteworthy that the Garcinia-based material achieved competitive dynamic performance despite its relatively moderate BET surface area (14.7 m^2^ g^−1^). This observation suggests that adsorption efficiency is not governed solely by surface area, but also by surface chemistry, functional group availability, and mass transfer behavior under column operation.

Direct comparison among studies must be interpreted cautiously, as experimental conditions—including influent concentration, bed dimensions, particle size, and breakthrough criteria—vary across investigations. Nevertheless, the results indicate that the Garcinia-derived adsorbent demonstrates performance comparable to several previously reported materials under fixed-bed conditions.

### Predicted adsorption mechanism of ammonium ions on Garcinia-based adsorbents

The adsorption of ammonium ions (NH_4_^+^) onto the Garcinia-derived material can be interpreted as a combination of surface interactions and mass transfer processes occurring under dynamic column conditions. Upon introduction of the influent solution into the packed bed, ammonium ions are transported from the bulk liquid phase to the external surface of the adsorbent through convective flow and external film diffusion.

FTIR analysis indicates the presence of oxygen-containing functional groups, primarily hydroxyl (–OH) and carboxyl (–COOH) moieties. These functional groups may participate in electrostatic attraction and ion-exchange processes. Under aqueous conditions, partial deprotonation of carboxylic groups can generate negatively charged surface sites, thereby enhancing affinity toward positively charged ammonium ions. Accordingly, electrostatic interaction and surface ion exchange are considered the principal adsorption pathways.

Following initial surface binding, gradual penetration of ammonium ions into internal pores may occur. SEM analysis reveals a heterogeneous and porous morphology, suggesting the existence of accessible diffusion pathways. However, SEM provides morphological evidence only and does not independently verify the adsorption mechanism. The progressive development of breakthrough curves further this suggests that intraparticle diffusion may contribute to the observed adsorption behavior.

The dynamic adsorption behavior is consistent with the assumptions of the Thomas and Yoon–Nelson models, which describe breakthrough evolution based on kinetic and mass-transfer-controlled processes. The agreement between experimental and predicted data suggests that adsorption is governed by coupled transport and surface interaction phenomena rather than instantaneous equilibrium. No regeneration experiments were conducted in the present study; therefore, reusability and long-term cyclic stability were not evaluated and require further investigation before practical implementation can be considered. Overall, ammonium removal by the Garcinia-based material appears to be predominantly controlled by electrostatic attraction and ion-exchange interactions associated with surface oxygenated functional groups, while mass transfer effects influence column performance under dynamic flow conditions.

To ensure the reliability of the presented results, all numerical values reported in the text were cross-verified with the corresponding tables and figures. Units were standardized (mg L^−1^ for concentration, min for time, cm for bed height, and mL min^-1^ for flow rate), and discrepancies were corrected to maintain full consistency across the manuscript.

## Conclusion

The performance of the Garcinia-derived adsorbent was evaluated under continuous fixed-bed column conditions for ammonium (NH_4_⁺) removal from aqueous solution. The experimental results demonstrated that adsorption behavior was influenced by operating parameters including influent concentration, flow rate, and bed height. An increase in initial ammonium concentration enhanced the mass transfer driving force but led to earlier breakthrough under dynamic conditions. Lower flow rates extended the residence time within the packed bed, resulting in delayed breakthrough and improved bed utilization. Increasing bed height shifted the C_t_/C_o_ profiles toward longer operational times, indicating greater adsorption capacity under extended contact conditions. The Thomas and Yoon–Nelson models adequately described breakthrough development and provided reasonable estimates of dynamic adsorption parameters within the studied operating range. The agreement between experimental and predicted data suggests that adsorption behavior under the investigated conditions can be interpreted using mass-transfer-based kinetic models. Physicochemical characterization confirmed the presence of oxygen-containing functional groups and a heterogeneous porous structure, which are consistent with electrostatic interaction and ion-exchange mechanisms proposed for ammonium uptake. The adsorption capacity obtained in this study is comparable to values reported for several bio-based and mineral adsorbents operating under fixed-bed conditions. However, variations in experimental design among different studies require cautious interpretation of direct comparisons. No regeneration experiments were conducted; therefore, long-term stability, cyclic performance, and economic feasibility remain to be evaluated. Further investigation including regeneration assessment and pilot-scale validation is necessary before practical implementation can be considered. Overall, the results indicate that Garcinia-derived material can function as a promising bio-based adsorbent under laboratory column conditions.

## Data Availability

The datasets used and/or analyzed during the current study are available from the corresponding author on reasonable request.
